# Temporal and Spatial Analysis of Neural Tube Defects and Detection of Geographical Factors in Shanxi Province, China

**DOI:** 10.1371/journal.pone.0150332

**Published:** 2016-04-21

**Authors:** Yilan Liao, Yan Zhang, Lei He, Jinfeng Wang, Xin Liu, Ningxu Zhang, Bing Xu

**Affiliations:** 1 The State Key Laboratory of Resources and Environmental Information System, Institute of Geographic Sciences and Natural Resources Research, Chinese Academy of Sciences, Beijing, 100101, China; 2 The Key Laboratory of Surveillance and Early-Warning on Infectious Disease, Chinese Center for Disease Control and Prevention, Beijing, 102206, China; 3 Institute of Agricultural Economics and Information, Henan Academy of Agricultural Sciences, Zhengzhou, 450002, China; 4 Department of Statistics, University of California, Berkeley, California, 94720, United States of America; 5 Department of Spatial Sciences, Curtin University, Bentley, WA, 6845, Australia; 6 Chang'an University, Xi’an, 710054, China; 7 Jiangsu Center for Collaborative Innovation in Geographical Information Resource Development and Application, Nanjing, 210023, China; Tabriz University of Medical Sciences, ISLAMIC REPUBLIC OF IRAN

## Abstract

**Background:**

Neural tube defects (NTDs) are congenital birth defects that occur in the central nervous system, and they have the highest incidence among all birth defects. Shanxi Province in China has the world’s highest rate of NTDs. Since the 1990s, China’s government has worked on many birth defect prevention programs to reduce the occurrence of NTDs, such as pregnancy planning, health education, genetic counseling, antenatal ultrasonography and serological screening. However, the rate of NTDs in Shanxi Province is still higher than the world’s average morbidity rate after intervention. In addition, Shanxi Province has abundant coal reserves, and is the largest coal production province in China. The objectives of this study are to determine the temporal and spatial variation of the NTD rate in rural areas of Shanxi Province, China, and identify geographical environmental factors that were associated with NTDs in the risk area.

**Methods:**

In this study, Heshun County and Yuanping County in Shanxi Province, which have high incidence of NTDs, were selected as the study areas. Two paired sample T test was used to analyze the changes in the risk of NTDs from the time dimension. Ripley’s k function and spatial filtering were combined with geographic information system (GIS) software to study the changes in the risk of NTDs from the spatial dimension. In addition, geographical detectors were used to identify the risk geographical environmental factors of NTDs in the study areas, especially the areas close to the coal sites and main roads.

**Results:**

In both Heshun County and Yuanping County, the incidence of NTDs was significantly (P<0.05) reduced after intervention. The results from spatial analysis showed that significant spatial heterogeneity existed in both counties. NTD clusters were still identified in areas close to coal sites and main roads after interventions. This study also revealed that the elevation, fault and soil types always had a larger influence on the incidence of NTDs in our study areas. In addition, distance to the river was a risk factor of NTDs in areas close to the coal sites and main roads.

**Conclusion:**

The existing interventions may have played an important role to reduce the incidence of NTDs. However, there is still spatial heterogeneity in both counties after using the traditional intervention methods. The government needs to take more measures to strengthen the environmental restoration to prevent the occurrence of NTDs, especially those areas close to coal sites and main roads. The outcome of this research provides an important theoretical basis and technical support for the government to prevent the occurrence of NTDs.

## Background

Neural tube defects (NTDs) are congenital birth defects that occur in the central nervous system. Among the birth defects, NTDs have the highest incidence, and its consequences are the most serious. In the early stages of embryonic period, the embryo is very sensitive and fragile. When it is influenced by genetic and environmental factors, it will cause neural tube closure not complete, thus forming anencephaly, spina bifida and encephalocele, etc [1]. NTDs are one of the main causes of abortion, stillbirth, stillbirth, and is also one of the main factors of infant deaths and permanently disabled. NTDs not only cause serious damage to the child survival and the quality of life, but also have an impact on the happiness and harmonious of a family. It can cause a heavy economic burden to family and society [2].

The cause of NTDs is complex. There has no definite conclusions about it, and now more unified view is that NTDs are caused by the environmental and genetic risk factors, or the complex interaction between them. NTDs is not reversible, and it is easy to happen when the embryonic development in 3~4 weeks and is exposed to adverse environment. Many environmental factors in the previous studies have been determined to be associated with the occurrence of NTDs, such as folic acid deficiencies [3], chemical exposure [4], radiation [5], soil type [6], and geophysical condition [7], etc.

China, a populous country, has the highest incidence of NTDs in the world [8]. While, Shanxi Province is in the peak of the incidence of NTDs in China [9]. Shanxi Province is also the largest coal production province in China [10]. There have been many studies on the relationship between environment and NTDs in Shanxi province so far. Previous studies found the watershed, lithozone, soil and distance to faults were closely related to the occurrences of NTDs [7, 8], while these studies were only a time cross sectional study. In addition, Gu et al [11] studied the spatial distribution of coal industry and NTDs, and displayed the interactive relationship of them. However, the driving factors of NTDs behind this phenomenon were not explored in previous studies. The temporal and spatial variation of NTDs prevalence and the impact from environmental risk factors in Shanxi need to be further analyzed.

One of the targets of studying the pathogen of NTDs is to make efficient intervention policy. NTDs intervention is a comprehensive and systematic project. Since 1999, the Chinese government had adopted a series of interventions such as the tertiary prevention measures to reduce the incidence of NTDs. The primary prevention measures are taken in pregnancy and the early stages of pregnancy, in order to prevent the occurrence of birth defects. The secondary prevention measures are put into practice during pregnancy of prenatal screening and prenatal diagnosis, in order to avoid serious birth defects. The tertiary prevention measures were applied after the birth of neonatal disease screening, in order to detect and treat diseases and exceptions in newborn babies early [8]. In 2004, Shanxi provincial Commission of Population and Family Planning established a birth defect intervention project called “Jianmao”, to reduce the occurrence of NTDs in Zhongyang, Jiaokou, Liulin, and Heshun Counties. The main objectives of the project are to reinforce the food fortification by adding five micro-nutrients: Vitamin B1, Vitamin B2, folic acid, iron and zinc in the flour and to make it accepted by local residents [12]. In 2009, Yuanping County adopted a similar intervention: distributing folic acid supplements to all the women in pre-pregnancy and with first trimester [13].

In this study, there were three research objectives: (1) to determine whether there was significant decline in the incidence of NTDs in Heshun County and Yuanping County? (2) to evaluate the spatial heterogeneity level after intervention in both counties? (3) to identify which were the specific environmental risk factors of NTDs in the study areas, especially some risk area?

## Materials and Methods

### Study areas

**Fig 1:**
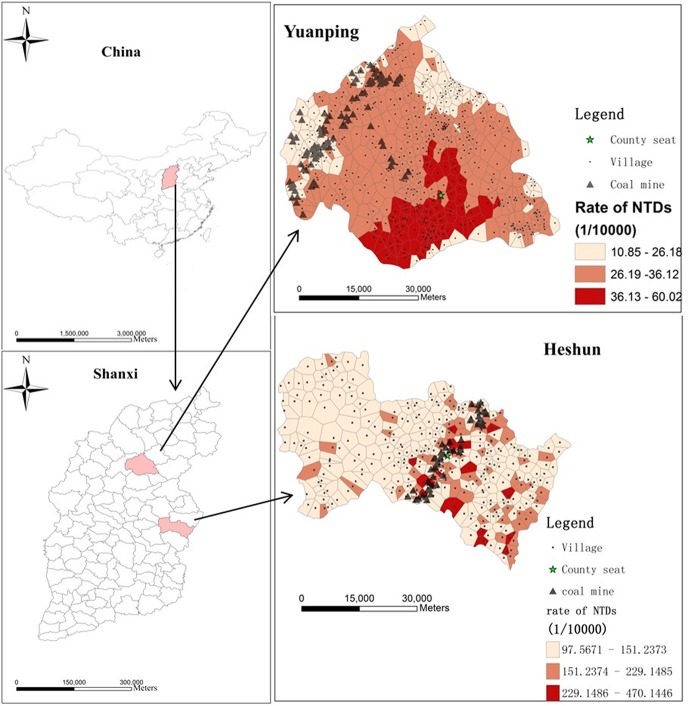
Location of study areas.

This study took place in two counties, which have high rate of NTDs in Shanxi province, Heshun County and Yuanping County, respectively (as shown in Fig 1.). Heshun County locates in the eastern of Shanxi Province, at 37°03'E and 113°05'N. Heshun lies in the middle of the Taihang Mountains. Therefore, its terrain is steep, mostly with mountains and hills. The climate is temperate continental climate. There are more than ten kinds of domestic mineral resources of coal, iron, aluminum, copper, etc. The coal reserves is about 3.5 billion tons. Heshun County has 326 administrative villages, and an area of 2,250 km^2^. There were 252 NTDs cases reported between 1998 and 2010. Correspondingly, there were 15030 births in in this period of time.

Yuanping County is located in the north of Shanxi Province, at 38°35' ~39°09' E and 112°17'~ 113°35'N. Yuanping County has 520 administrative villages, with an area of 2560 km^2^. Yuanping County is an important commodity grain production base and vegetable production base of Shanxi. Compared with the Heshun County, the economic condition of Yuanping County is relatively better. 107 NTDs cases were reported from 2007 to 2012 period. Correspondingly, there were 20215 births in this period of time.

Heshun County is the key coal production base in Shanxi Province. Heshun County has a coal bearing area of 1,852 km^2^, which accounts for 82.3% of the county’s total area. The county’s coal bearing area contains total reserves of 156 billion tons, of which 34 billion tons have been identified. The coal production in Heshun County is 8 million tons per year. Heshun County had about 80 coal sites according to the materials provided by the Heshun Bureau of Coal Industry. While Yuanping County has a coal bearing area of 400 km^2^, which accounts for 15.6% of the county’s total area. The county’s coal bearing area contains total reserves of 1.12 billion tons. Yuanping County had about 14 coal sites according to the materials provided by the Yuanping Bureau of Coal Industry. The coal output there is 4 million tons per year. It noted that Yuanping County is one of the key transfer stations of China’s main coal transportation railway.

### Data sources

In the study, the study period of Heshun County is from January 1, 1998 to December 31, 2010, while the study period of Yuanping County is from January 1, 2007 to December 31, 2012. The study included all live and still births occurring in Heshun County and Yuanping County in the study period (as listed in S1 File and S2 File), born to women at the hospital or at home, and who were residents of the counties during those time periods. All therapeutic abortions in residents in those areas where the estimated date of delivery fell in the time periods of interest were all included. All NTDs cases, regardless of pregnancy outcome, were verified by doctors in the hospitals. All of the cases, regardless of pregnancy outcome, were provided by the local family planning department. The NTDs in the study included anencephaly, spina bifida, and encephalocele, hydrocephalus, etc. All cases were location to villages except unable insured cases.

Since the Local Family Planning Department declined to provide identifiers to link substantiated NTDs cases to births, so we carried out an ecological study instead of studying at the individual level. It means that we should use the NTDs cases and the villages’ environment where patient’s mother had been living to estimate the risk of NTDs. However, towns in those two counties were not included in the study as the environmental factors there are somewhat complex. Moreover, birth defect registers in the town were excluded.

We assumed that the intervention projects in Heshun County and Yuanping County respectively began to show effects by 2004 and 2009. For the purpose of calculating the occurrence ratios before and after intervention, the reported NTDs cases in Heshun County during the 1998–2004 period and during the 2005–2010 period were combined. In the same way, the NTDs of Yuanping County were divided into the 2007–2009 year cases and the 2010–2012 year cases.

The statistical unit of socio-economic data is not at the village level, which is inconsistent with the geographical environment data, therefore, this study only analyzed the relationship between geographical environment factors and NTDs. According to previous studies, the geographical environmental factors mainly include elevation, soil type, land-cover types, water shortage condition (distance to rivers), and geological background (distance to faults) [8]. The information of the elevation and land-cover type used in this paper was from the geospatial data cloud (http://www.gscloud.cn/). ArcGIS 10.0 was used to store geographic data and display the results of this study.

The Biomedical Ethics Committee of Peking University approved this retrospective study. Because our study is ecological study, the detailed information of patients are not included in the study. Moreover, all patient records/information was anonymized and de-identified prior to analysis. So we did not give written informed consent to participants (or next of kin/caregiver in the case of children) for their clinical records to be used in this study.

### Methodology

In the study, we first used two paired sample T test to study the temporal changes in the risk of NTDs. Ripley’s K function and spatial filtering were then combined with GIS software to determine the spatial changes of the NTDs risk. Finally, geographical detectors were applied to identify the risk factors of geographical environmental for NTDs in the study areas, especially the area close to the coal sites and main roads.

#### Traditional statistical analysis

Two paired sample T test is a statistical method that compares the population means between two related groups. Normally, two paired sample T test is used to test whether there is significant difference before and after an experiments [14]. In this study, two paired sample T test was applied to compare the difference of NTDs averages in different periods. The data were statistically analyzed using SPSS Win 19.0, and a 95% level of significance was applied.

#### Ripley’s K function

Ripley’s K function is a classical tool to analyze the spatial structure of point process. It can be used to determine whether the point data is clustered at different spatial distances. In the Ripley’s K function, the K-function is compared with a homogeneous Poisson distribution. The formula is written as (following Durbeck et al. [15]):

$$\begin{array}{l} L\left(h\right)=\sqrt{\frac{K\left(h\right)}{\pi }}\\ K\left(h\right)=\frac{R}{n^{2}}\sum_{i=1}^{n}\sum_{j=1,i\neq j}^{n}\frac{I_{h}\left(d_{ij}\right)}{w_{ij}} \end{array}$$ (1)

Where *R* is the area of the study region, *n* is the total number of NTDs in the study region, *d*_*ij*_ is the distance between villages *i* and *j*, *I*_*h*_(*d*_*ij*_) is j an indicator that equals 1, if *d*_*ij*_ is less than *h*, and equal to 0 otherwise. *w*_*ij*_ is an edge correction factor and defined from 0.5 to 1.

Ripley’s K function in this study was used to test whether there was a global spatial autocorrelation in the distribution of NTDs in Heshun County and Yuanping County, respectively. After that, spatial filtering, a method of local spatial autocorrelation, was applied to detect the hotspots of NTDs in both two counties.

#### Spatial filtering method

Spatial filtering method uses non-parametric statistical techniques as a tool for exploratory spatial data analysis [16]. The main purpose of spatial filtering method is to estimate disease risk and conduct statistical tests to detect high risk spots of certain disease within study area. It provides a way to determine the lack health care access, potential environmental and behavioral elements. It has also used to identify clusters of congenital malformation, infant mortality, and other mortality of birth disease [17, 18, 19].

In this study, the Distance Mapping and Analysis Program (DMAP), statistical software, was used for cluster analysis. It is a public- free software package and can be downloaded from www.uiowa.edu/~gishlth/DMAP4/, which uses the method of Rushton spatial filtering. The Rushton spatial filtering method first used regular grid points as the center, which covering the study area, to generate a series of filtering circles [20]. For each circle, the rate was calculated as crude ratio of NTDs cases to all births. The center of each grid can be considered as sharing the same value of the circle it falls in. Therefore, the distribution of NTDs rate can be interpolated as a continuous space. The neighbor grid points share overlapping circular pattern, thus sharing the observed information. After the estimated rate was assigned to the grid points, a contour map was constructed by GIS [20]. We supposed that the probability of a case resulting to NTDs is equal to the proportion of all births in the regions where resulted in NTDs. A ‘smoothed’ morbidity map was drawn where has significance levels of high morbidity of NTDs by percentage for all individual circle were calculated and mapped in an isarithm form. The analysis of DMAP provides us many valuable information, such as the rate NTDs and statistical significance of observed NTDs rates in each grid. The hot spots can be identified from the grids where significance was higher than the threshold value.

#### Geographical detectors

Geographical detectors were presented by Jinfeng Wang in 2010, using spatial variance analysis to quantitatively descript the relationship between risk factors and certain disease [7]. The geographical detectors include four probes: risk detector, factor detector, ecological detector and interaction detector. Risk detector can identify the areas with health risk and the category partition which has higher health risk and test significance by comparing the average of the health risk index with different category partitions. Factor detector can determine whether a certain geographical factors is associated with certain spatial distribution pattern of health risk. It explore risk factors by comparing the total variance of health risk index in different category partition, with the total variance of health indicators in the entire study area. The smaller the ratio, the larger impact the factors have. Ecological detector compares the difference in the total variance of health risk index between the different elements. It evaluates whether there is a significant difference of the impact on the spatial distribution of disease between different geographic factors. Interaction detector can identify the interaction influence between risk factors. This method not only considers the consistency and differences between the disease and nature, human elements on the spatial distribution, but also is applicable to both observed data and categories data [7]. Geographical detectors have been widely used in the areas of birth defects [7], earthquake death [19], ecology [20] and urbanization [21].

The influence of factors on NTDs in this study was calculated by the Power of Determinant (PD):

$$PD=1-\frac{1}{N\sigma^{2}}\sum_{i=1}^{L}N_{i}\sigma_{i}^{2}$$ (2)

Where N is the area of the study, *σ*^2^ is the variance of disease rate in the study area. The study area is divided into L stratums, and expressed as *i* = 1,2,3,…,*L* [22], based on the spatial heterogeneity of a suspected factor. Usuallys, if the factor completely controls the disease, PD equals to 1; and if the determinant is completely unrelated to the disease, PD equals to 0. Thus, the PD reflects the association level between risk factors and the prevalence of the disease [7].

## Results

### Statistical analysis

The addresses of recorded cases and births were matched to the village points using ArcGIS desktop. The two paired sample T test results (Table 1) showed that the average NTDs rates was reduced significantly (P<0.01) in both counties. The incidence of NTDs in Heshun County has been reduced from 233.1065 per 10,000 births before intervention to 105.9737 per 10,000 births after intervention in Heshun County. While, the incidence of NTDs in Yuanping County has been reduced from 41.0998 per 10,000 births before intervention to 24.7762 per 10,000 births after intervention.

**Table 1:** Two paired sample T test results of the average rate of NTDs before and after intervention in the study areas.

Location	average rate of NTDs before intervention (1/10000)	average rate of NTDs after intervention (1/10000)	t value	degrees of freedom	p value
Hshun	223.107	105.974	31.442	325	<0.001
Yuanping	41.100	24.776	42.454	519	<0.001

### Spatial analysis

#### Global autocorrelation analysis

In order to testify whether the distribution of NTDs in Heshun County and Yuanping County is clustered, we applied Ripley’ s K function on both of them. The following graphs are the results of Ripley’s K function in three periods of Heshun County respectively: from year 1998 to year 2004 (before the intervention), year 2005 to year 2010 (after the intervention) and year 1998 to year 2010 (the whole observation period). Similarly, the results of Yuanping County from Ripley’s K function were: from year 2007 to year 2009 (before the intervention), year 2010 to year 2012 (after the intervention) and year 2007 to year 2012 (the whole observation period).

**Fig 2:**
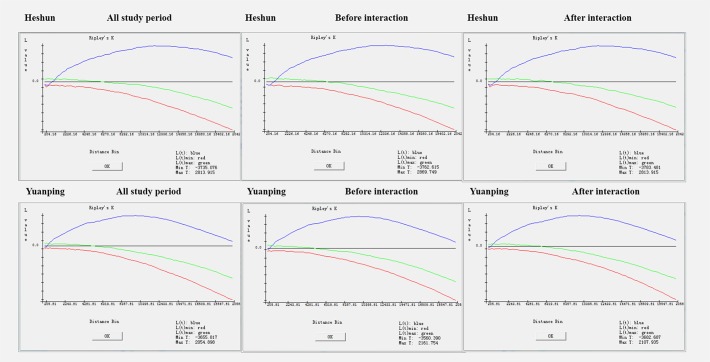
The results of Ripley’s K function of NTDs in Heshun County and Yuanping County.

Overall, the results above suggest that the distribution of NTDs in Heshun and Yuanping was clustering in all three periods. From Fig 2 (blue lines; average levels), the spatial cluster of NTDs in Yuanping can be identified from a very small distance (nearly zero meter) for all the study periods. In contrast, at a relative small distance, a spatial negative aggregated was identified in Heshun for all the study periods. This relationship changes to positive at distance about 210 m. The largest positive relationships were found at distance about 12–13 km and about 10 km in Heshun and Yuanping, respectively (Table 2). While, decreases of the cluster level were followed beyond these distances in both study areas.

**Table 2:** The largest extent of clustering area in three periods in Heshun County and Yuanping County.

	Heshun County		Yuanping County	
	The Largest L value	Corresponding Distance (kilometer)	The largest L value	Corresponding Distance (kilometer)
Before the intervention	2869.750	13.066	2161.754	10.084
After the intervention	2813.915	12.045	2107.935	10.084
The entire period	2813.915	12.045	2054.898	10.084

#### Hotspot detection

By matching NTDs case and birth records to villages, it is able to count NTDs rates for each grid, which covers the entire study area at about 4.8km intervals. In order to come to general conclusions about the results of spatial filtering, multiple filter sizes such as 1.6, 3.2, and 4.8 km were used. We carried out a sensitivity analysis using different spatial filter sizes, the results are shown Table 3.

**Table 3:** Sensitivity analysis results based on different spatial filter sizes.

Filter Size	Number of clusters	Number of grids included in clusters
1.6 km	3	3
2.4 km	2	4
3.2 km	2	4
4.0 km	2	9
4.8 km	1	15

When the filter size increases to 4.8 km, the number of clusters decreases to 1 and the number of grids included in clusters increases to 15. The result suggests that progressively larger spatial filtering of data removes local spatial variability, which eventually produces a substantially uniform pattern. Moreover, more grids included leads to more reliable results.

Given the approximate 4.8km distance interval between grid intersections, it generated 204 grid points in Heshun County and 326 grid points in Yuanping County. Meaningful NTDs rates were estimated for these grid points which had at least 5 births within the 4.8km vicinity. We calculated the NTDs and normal births within the circle and assigned the observed rate to that point. It is able to interpolate the NTDs rate as a continuous spatial distribution, when we repeated such estimate for a grid. Neighbor grid points shared circular patterns which overlap each other. Therefore, they share observations. Isarithm maps with the constant range of values were constructed in GIS after the estimated rates were assigned to grid points.

For each village, a random number was generated from a uniform distribution in the range of 1 through 1,000. For each grid point locations, 999 Monte Carlo simulations were run and the different NTDs rates were rank-ordered. The percent of the simulated rates at each grid, that were less than the observed rate for the same grid location, were calculated and the levels of statistical significance were portrayed as isoclines.

**Fig 3:**
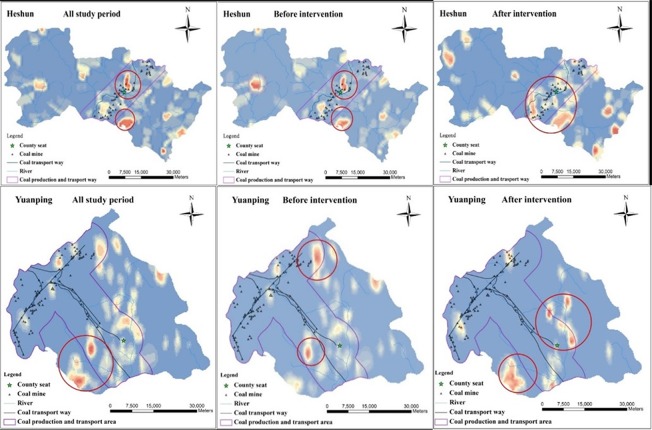
Cluster areas of NTDs in Heshun County and Yuanping County.

Because the testing of rates against 999 simulations is one type of exploratory spatial analysis, the methods of representing the results were discretionary, and the results can be adjusted by investigators based on the level of significance. Fig 2 denotes the clusters of NTDs in Heshun County during 1998–2010 and Yuanping County during 2007–2012. The results (seen in Fig 3) showed that clusters of NTDs were detected in Heshun County and Yuanping County during the whole study period, before and after intervention, which showed that the spatial distribution of NTDs in Heshun County was nonrandomized. Compared to the period of before intervention, the radius of cluster was become bigger in Heshun County and Yuanping County. Although the location and size of NTDs clusters after intervention were changed after intervention, clusters of NTDs were always existed nearby the roads for coal transport in Heshun County and Yuanping County.

### Geographical detectors

In Heshun County, 6 km buffer region of coal mines and main road was defined as the coal production and transport area. This is because the rate of NTDs in these regions was significant higher than other region in Heshun County [8]. We adopt a buffer step analysis in Yuanping County, and found the rate of NTDs in the region within 8 km buffer distance of the roads for coal transport was significant higher than other region of Yuanping County (P = 0.007). Therefore, 8 km buffer region of coal sites and roads for coal transport was defined as the coal production and transport area in Yuanping County. The selected ranges of buffer regions are consistent with the results of Ripley’ s K function.

According to the result of Fig 2, we can find the clusters of NTDs were always existed in the coal production and transport area in Heshun County and Yuanping County. We also conducted a detection of geographical environment factors for NTDs in the two counties, especially in the coal production and transport area. The influence of different geographic environmental risk factors on NTDs in different areas were showed by PD in below (Tables 4 and 5).

**Table 4:** Factor detector results of NTDs in the whole study region.

Location	Period	Factor				
		Elevation (%)	Fault (%)	River (%)	Soil (%)	Land-cover (%)
Heshun	All	2.5	3.7	4.2	6.1	0.4
	Before	3.4	4.7	2.2	7.4	0.3
	After	2.5	2.2	2.7	6.8	2.4
	Trend	↓	↓	↓	↓	↑
Yuanping	All	16.3	14	6.2	16.8	3.9
	Before	6.3	22.2	8.5	4.7	2
	After	12	17	5.2	8	6.3
	Trend	↑	↓	↓	↑	↑

**Table 5:** Factor detector results of NTDs in coal production and transport area.

location	Period	Factor				
		Elevation (%)	Fault (%)	River (%)	Soil (%)	Land-cover (%)
Heshun	All	48.6	19.4	14.5	5.4	4.1
	Before	24.8	12.7	15.2	5.5	3
	After	69.5	22.4	13.4	4.4	3.3
	Trend	↑	↑	↓	↓	↑
Yuanping	All	35.8	34.9	14.5	17.5	10.4
	Before	40.2	54.6	15.2	20.4	10
	After	40.2	49.3	13.4	23.8	14.1
	Trend	—	↓	↓	↑	↑

Over the entire period, the results of the factor detector suggests that the impact on the NTDs incidence of different environmental factors were different. In Heshun County, the descending order of the impact is: soil type (6%), distance to the coal sites and transportation region (4.7%), river (4.2%), fault (3.7%), elevation (2.5%), land cover (0.4%). In Yuanping County, the descending order of the impact is: soil type (16.8%), elevation (16.3%), fault (14%), river (6%), coal sites and transportation region (4.6%), land cover (3.9%). By comparing the two orders above, it found that the first four determinates are all natural environmental factors in Yuanping. It means that the natural environment has greater influence on the NTDs incidence. In Heshun, although the impact of natural environmental factors is still considerable, the difference is that coal site and transportation region has the second largest impact on the NTDs incidence. It shows that the impact of coal mining and transportation, which is regarded as human behavior, has higher correlation with the NTDs incidence in Heshun. In addition, soil type is the most correlated environmental factor of the NTDs incidence in both Yuanping and Heshun. Combined with analysis results of the risk detector, the area of cinnamon soil in both Heshun and Yuanping has relatively higher NTDs incidence.

By comparing the results from factor detector of pre-intervention and post-intervention period, the impact from some environmental factors in Yuanping and Heshun has diminished significantly. In Yuanping County, the PD values of fault, river and buffer distance have reduced from 22% to 17%, 8% to 5% and 2.8% to 0.5%, respectively. In Heshun County, the PD values of buffer distance and fault have reduced from 4.9% to 1.9% and 4.6% to 2.2% respectively.

After the intervention, the rankings of the impact of the selected environmental factors have changed. In Yuanping County, fault, elevation and soil type have relatively high correlation with the incidence of NTDs. Combined with the result from the risk detector, it indicates that the distance to the fault is smaller, the NTDs incidence will be higher. In addition, the lower elevation area tends to have higher incidence of NTDs. Furthermore, the area of leached cinnamon soil has relatively lower NTDs incidence. In Heshun County, soil type remains the most related factor of the five we selected. However, the only difference is that each type of the soil has very similar correlation with the incidence of NTDs. It suggested that the variation of impact from different soil types becomes insignificant.

In coal production and transport area, the impact from the potential factors are more apparent and significant in both study areas. In Yuanping County, the impact analysis results show there wasn’t a significant variation before and after intervention, and the impact order from different factors didn’t change: elevation (35.8%)>fault(34.9%)>soil(17.5%)>river(14.5%)>land-cover(10.4%). While, it did change in Heshun. In coal production and transport area, the impact from factors related to soil type decreased from 6.1% to 5.4%, and the impact from elevation significantly increased from 2.5% to 48.6%.

## Discussion

The incidence of NTDs was significantly reduced after intervention in both Heshun County and Yuanping County, Shanxi Province. The average incidence of NTDs in Heshun County and Yuanping County is higher China average morbidity (12.95 per 10,000) [23], and spatial heterogeneity still exists in study areas. This study also found that the clusters of NTDs always existed in the coal site zones and transport area in Heshun County and Yuanping County. This study shows that the intervention may have a positive impact on reducing the overall incidence of NTDs.

China's birth defects monitoring survey figures show that the incidence of NTDs in the eight major coal producing areas in Shanxi Province is much higher than the national average [24]. Heshun County and Yuanping County are the key coal-producing counties in Shanxi Province. Most of these coal sites are small town and village owns or illegal coal sites before Shanxi coal enterprises integrate and restructure them [25]. There are some trace elements in coal, such as mercury, nickel, cadmium, fluoride, etc [26]. Coal mining and coal washing will also produce large amounts of dust particles, and this will pollute the water [27]. The pollution was prevented by coal enterprises, however, there have no ecological restoration be carried out, and therefore the hazard of pollution still exists in coal production and transport area. In our study, the clusters exist in coal production and transport area, which were detected by spatial statistic, also support this view.

It is very important to identify the geographical environmental risk factors of NTDs in the study area, especially in the coal production and transport area. Based on the results from geographical detectors, the government can develop preventive measures for people living in these regions. For example, elevation may be a high risk factor of NTDs, previous studies shown that altitude may be as a risk factor for NTDs [28]. The incidence of NTDs in high altitude areas is higher than other area in coal production and transport area. It’s very necessary to learn the surrounding of villages with NTDs case. Generally, the limited medical condition and restricted access to information of interventions in high elevation area reduced the efficiency of the implementation of interventions [8]. Also, traffic in high altitude mountain areas is inconvenience. Therefore, women cannot go to the hospital for NTDs prenatal screening after pregnant, thus delaying the best treatment period of NTDs [25].

In addition, people living near a geological fault experience a high risk of having a baby with NTDs [29]. There is a significant difference of the NTDs incidence between the areas close to fault and other regions. Previous studies have shown that some heavy metals in coal are likely to cause the occurrence of NTDs [30]. During the coal production and transport process, heavy metals in coal will be exposed and pollute the groundwater, when they flow in the underground passages which was formed by fault. Fault is related to the source of environmental chemicals, and act as a source of heavy metals to influence the occurrence of NTDs [29]. Moreover, the concentration of radon in soil, water, and air near a fault zone is much higher than the background value. The radiation emitted by radon and the daughter products into which it decays is predominantly high linear energy transfer (LET) alpha radiation. For any given absorbed dose of radiation, the relative biological effectiveness (RBE) of alpha radiation as emitted by radon and its daughters is 20-fold higher than that of X-ray and gamma radiation. The fetus in uterus is at particular risk of damage from radiation [31]. Thus it may lead to the higher NTDs incidence in fault zones. In addition, the soil as carrier of toxic substances have an impact on the incidence of NTDs in the coal production and transportation area [32]. Cinnamon soil has high contents of metals, which may have an influence on human health and PH value is 7.0–7.5. In soil with high organic matter content and pH value, metals usually form complexes and thus cannot easily be absorbed by crops. This finally affects the diet of local residents [33]. Leached cinnamon soil has high contents of clay particles. Theoretically, the more clay particles percent content, the larger adsorption capacity, and then smaller amounts of health-related metals can be absorbed by plants and do harm, therefore, there is less danger to local residents [34]. However, these inferences need further analysis to be confirmed.

Coal mining operations are viewed as a widespread water pollution resource [31]. This study shows that the incidence of NTDs near river is significantly different (P<0.05) from other areas. The activity of heavy metal elements and rare earth elements in soil there may significantly improve the incidence of NTDs [35]. Soil is an important medium of various substances in the environment. It has impact on the content of toxic chemicals in soil, such as lead, mercury and other heavy metals, radioactive substances, nutrients, trace elements, etc. It also affects the activity of these chemicals in soil, thus affecting the chemical content in human living environment, and increasing the risk of NTDs in the those areas.

Coal production and coal transportation have significant impact on the heavy metal content in soil. Some heavy metals accumulate in the soil and has already achieved a higher level, especially the accumulation of heavy metals in the plow layer. Those metals may have adversely impact on crop, human and animal health [36]. Further research is required to study the accumulation and distribution of relevant heavy metal close coal sites and areas along the roads to deliver coal.

The impact of land cover on occurrence of NTDs becomes more significant recently. During the period of coal production and transport, the truck traffic loads are heavy. Overload big tonnage transportation vehicles increases yearly, and the pollution in those regions is very serious. More coal sites are developing now. Plants nearby are covered with black pulverized coal, and the exterior of the buildings nearby is exposing to the pollution. This has caused serious ecological destruction. It is a pressing problem for the government to take effective measures and strengthen the management to protect the ecological environment that has been polluted by coal industries. Then, this will reduce the incidence of NTDs fundamentally.

## Conclusions

The existing interventions have played an active role to reduce the incidence of NTDs, however, there are still existing spatial variation of NTDs. Although this study was only conducted in a small spatial scale, the analysis results can be used as reference for the Chinese government. The study demonstrates that it is necessary to take different interventions in different regions. In addition to the existing three-level intervention measures, the government needs take more measures to improve penetration of interventions in the areas close to coal sites and coal delivery routes. Furthermore, strengthen the environmental restoration of the coal production and transport area is the key to prevent the occurrence of NTDs.

There are some limitations in the research. Firstly, we only collected data at hospital level, which means our data do not include home births. Secondly, our research is limited to ecological analysis. This is because the individual level information is not available. Therefore, the environmental exposure of individual level cannot be assessed. It is necessary to identify the risk factors for each NTDs in future studies. Thirdly, a comparative study for the areas with coal exploitation and low NTDs incidence wasn’t implemented, due to the limited data resources. This topic could be covered in the future study, when the data is available.

## Supporting Information

S1 File The case and birth data of Heshun County. (XLS)

S2 File The case and birth data of Yuanping County. (XLS)
